# 3,1-Benzothiazines, 1,4-Benzodioxines and 1,4-Benzoxazines as Inhibitors of Matriptase-2: Outcome of a Focused Screening Approach

**DOI:** 10.3390/ph9010002

**Published:** 2016-01-13

**Authors:** Polya G. Roydeva, Anna-Madeleine Beckmann, Marit Stirnberg, Jožko Cesar, Danijel Kikelj, Janez Ilaš, Michael Gütschow

**Affiliations:** 1 Pharmaceutical Chemistry I, Pharmaceutical Institute, University of Bonn, An der Immenburg 4, D-53115 Bonn, Germany; polq_roideva@abv.bg (P.G.R.); annabeckmann@web.de (A.-M.B.); marit.stirnberg@uni-bonn.de (M.S.); 2 Faculty of Pharmacy, University of Ljubljana, Aškerčeva cesta 7, SI-1000 Ljubljana, Slovenia; Jozko.Cesar@ffa.uni-lj.si (J.C.); Danijel.Kikelj@ffa.uni-lj.si (D.K.); Janez.Ilas@ffa.uni-lj.si (J.I.)

**Keywords:** benzamidines, 4*H*-3,1-benzothiazin-4-ones, 2,3-dihydro-1,4-benzodioxines, 3,4-dihydro-2*H*-1,4-benzoxazines, matriptase-2, protease inhibition

## Abstract

The liver enzyme matriptase-2 is a multi-domain, transmembrane serine protease with an extracellular, C-terminal catalytic domain. Synthetic low-molecular weight inhibitors of matriptase-2 have potential as therapeutics to treat iron overload syndromes, in particular in patients with β-thalassemia. A sub-library of 64 compounds was screened for matriptase-2 inhibition and several active compounds were identified. (*S*)-Ethyl 2-(benzyl(3-((4-carbamidoylphenoxy)methyl)-2,3-dihydrobenzo[*b*][1,4]dioxin-6-yl)amino)-2-oxoacetate ((*S*)-**12**) showed an IC_50_ value of less than 10 µM. Structure-activity relationships were discussed and proposals to design new matriptase-2 inhibitors were made.

## 1. Introduction

Thalassemias are among the most common inherited diseases worldwide. They are classified as anaemia and typified by abnormal formation of hemoglobin [1]. One type of disease, β-thalassemia, is characterized by a decreased synthesis of β-globin chains or by the complete lack of it, resulting in a severe anaemia and/or red blood cell abnormalities. The imbalance between the amount of α- and β-globin chain leads to extra medullary expansion and splenomegaly [1]. Patients affected by β-thalassemia major, the most severe form, require chronic red blood cell transfusions. As a result, they develop secondary iron overload. The milder form, β-thalassemia intermedia, does not necessitate blood transfusions, but also leads to iron overload due to chronic suppression of the hepcidin synthesis caused by ineffective erythropoiesis, thereby leading to increased iron absorption in the duodenum [1,2,3,4]. Untreated iron overload causes liver cirrhosis, cardiomyopathy, diabetes, arthritis, hypogonadism, and skin pigmentation and is the main reason for death in these individuals. In other diseases that are correlated with primary iron overload, like HFE-associated hemochromatosis, iron accumulation is hindered by phlebotomy, but this is not possible in the case of β-thalassemia. Therefore, patients must be treated with iron chelation therapy, in most cases through the subcutaneous application of desferoxamine.

Hepcidin, a small hepatic peptide hormone, has a crucial role in iron homeostasis in the human body [2,3]. Hepcidin negatively regulates intestinal iron absorption, iron recycling from macrophages and iron release from hepatic stores and macrophages into the plasma [3,5]. The expression of hepcidin is regulated by the BMP-SMAD pathway. Bone morphogenetic proteins (BMPs) are part of the transforming growth factor-β superfamily of ligands [3,4]. Two factors play a crucial role in the iron homeostasis, BMP6 and hemojuvelin. Hemojuvelin is a glycophosphatidylinositol-membrane-anchored co-receptor, detected mostly in hepatic cells [3,4]. It uses the BMP type I receptors ALK2 and ALK3 to transfer signals as a response to BMP6 [6]. The ligand BMP6 and the BMP-receptor complex activate the SMAD1,5,8/SMAD4 (sons of mother against decapentaplegic) complex. SMAD4 is translocated to the nucleus, where it is recognized by the hepcidin promoter [3,6], which is responsible for the hepcidin expression.

Matriptase-2, encoded by the gene *TMPRSS6*, known as a member of the type II transmembrane serine protease family, is located mainly at the cell surface of hepatocytes. The structure of this enzyme contains a cytoplasmic N-terminal domain, a transmembrane domain, a SEA (sea-urchin sperm protein, enteropeptidase and agrin) domain, two CUB (complement factor C1s/C1r, urchin embryonic growth factor, bone morphogenetic protein) domains, three LDLRA (low density lipoprotein receptor class A) domains and a C-terminal serine protease domain [7,8,9,10,11]. 

**Figure 1:**
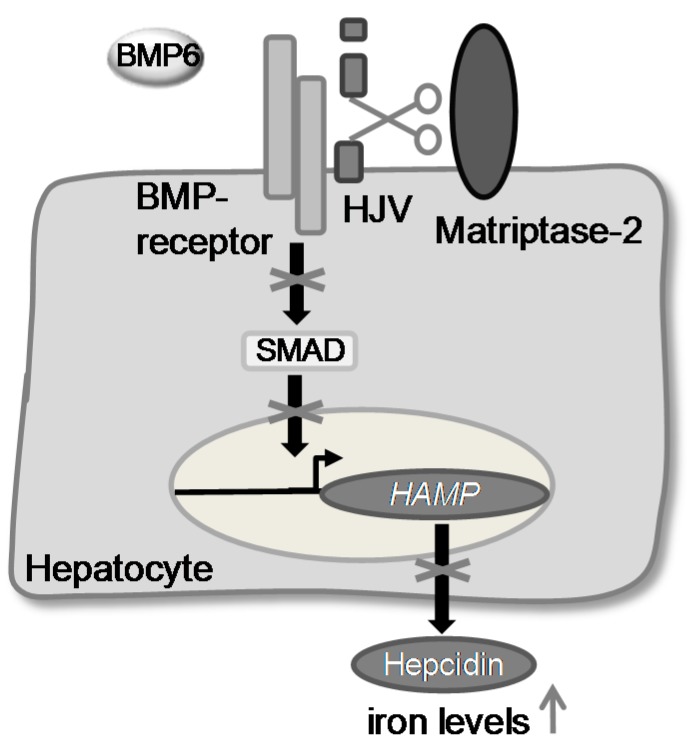
The postulated role of matriptase-2 in iron homeostasis.

Recently, it was shown that matriptase-2 represents a key enzyme in iron homeostasis [12,13,14]. Mutations in the *TMPRSS6* gene were found to cause iron-refractory iron deficieny anaemia (IRIDA) [17]. It was demonstrated that matriptase-2 acts as a suppressor of the expression of the hepatic hormone hepcidin. It probably inactivates the bone morphogenetic protein co-receptor hemojuvelin (m-HJV) by cleaving it into an inactive form [10,15]. As a consequence, the phosphorylation of SMADs (sons of mothers against decapentaplegic homologue) is suppressed and therefore the expression of *HAMP*, the gene encoding hepcidin, decreases. This leads to a higher level of iron in the blood plasma (Figure 1).

Several lines of evidence indicate that matriptase-2, as a trypsin-like serine protease, has a specificity to cleave the peptide bond after basic amino acids. For example, putative cleavage sites in hemojuvelin as well as autoprocessing cleavage sites feature arginine in the P1 position [10,11,15,16,17]. A preferred P4–P1 substrate sequence (Ile–Arg–Ala–Arg), obtained by a combinatorial approach, confirmed this primary substrate specificity [18], which is facilitated by the negatively charged aspartyl side chain at the bottom of their S1 pocket, able to interact with positively charged moieties, e.g., of arginine or arginine mimetics. Moreover, the S3/S4 region of matriptase-2 has also been found to be occupied by positively charged ligand moieties [18,19,20].

Inhibitors of matriptase-2 have potential as therapeutic compounds to treat iron overload syndromes, which are present in β-thalassemia patients [21,22,23,24]. Therefore, matriptase-2 is a promising pharmaceutical target for the development of synthetic inhibitors [10]. Meanwhile, first reports on low-molecular weight inhibitors of matriptase-2 have appeared, including dipeptide amides with a amidinobenzylamide residue [25], amidinophenylalanine derivatives [26], peptidic ketones [27], and sunflower trypsin inhibitor-1 analogues [28]. Moreover, bis- and trisbenzamidines [19,20] have been reported as matriptase-2 inhibitors, so former type of compounds also being valued as antiprotozoal and antifungal agents [29,30,31]. This study attempted to provide further heterocyclic, non-peptidic matriptase-2 inhibitors. By taking the primary substrate specificity of matriptase-2 into account, a focused screening approach was used and is described herein.

## 2. Results and Discussion

A sub-library of 64 compounds was evaluated for inhibition of matriptase-2. Structures of relevant compounds are shown in Table 1 and Table 2. Two classes were identified which include active compounds. The first class comprises 4*H*-3,1-benzothiazin-4-ones. The corresponding data are listed in Table 1. 4*H*-3,1-Benzothiazin-4-ones have previously been reported to exhibit dual activities as adenosine receptors antagonists and inhibitors of monoamine oxidase B [32,33]. Moreover, certain members act as oxoeicosanoid receptor ligands [34]. The structure of 4*H*-3,1-benzothiazin-4-ones can be considered to result from a bioisosteric replacement of the ring oxygen by sulfur. The oxygen analogues, 4*H*-3,1-benzoxazin-4-ones, have attracted attention as inhibitors for serine proteases, for example for human leukocyte elastase or chymase [35,36], but are less stable against unspecific nucleophiles than the 4*H*-3,1-benzothiazin-4-ones [37].

As a first representative of matriptase-2 inhibiting 4*H*-3,1-benzothiazin-4-ones, we identified compound **1** (Table 1). This molecule contains a basic side chain at position 6 of the heterocyclic skeleton. It was assumed that this basic group might be able to interact with the S1 pocket of matriptase-2. Thus, all 4*H*-3,1-benzothiazin-4-ones available in our library were considered and those compounds were selected which bear a basic residue at optional positions of the heterocyclic scaffold. Derivatives closely related to **1** were, however, found to be inactive. For example, a shift of the residue at position 6 to position 7 led to a loss of activity (**1** *versus* **2**). Compound **5** with an extended 6-residue and the more embedded basic nitrogen was also inactive, as the positively charged group might be prevented from interacting with the S1 pocket. We have evaluated three 4*H*-3,1-benzothiazin-4-ones which bear a basic moiety within the 2-substituent. While the presence of a primary amine structure in **7** yielded a second, active compound, derivatives **8** and **9** with tertiary amine substructures were inactive.

The second class of test compounds from which we have identified active representatives mainly consists of heterocycles which exclusively contain a benzamidine moiety. The benzamidine group is known to be efficiently accommodated in the S1 pocket of trypsin-like serine proteases. Benzamidine itself was also tested in the course of this study as an inhibitor of matriptase-2, but exhibited only weak activity with an IC_50_ value of more than 400 µM. The structures of the benzamidine-containing heterocycles and their IC_50_ values for matriptase-2 inhibition are outlined in Table 2. The first five entries include 2,3-dihydro-1,4-benzodioxines ((*S*)-**10** to (*S*)-**12**). These members have previously been reported to exhibit a dual activity, against thrombin and the fibrinogen receptor α_IIb_β_3_, with inhibition of the latter target producing an anti-platelet activity. Besides thrombin, activity against related serine proteases, e.g., trypsin and factor Xa, has also been identified. The enantiomers (*S*)-**10** and (*R*)-**10** as well as (*S*)-**11** and (*R*)-**11**, represent 6- and 7-substituted isomers [38].

Compounds **13**–**21** are racemic 3,4-dihydro-2*H*-1,4-benzoxazine derivatives with an oxymethylene spacer connecting the heterocyclic core with a *para*-benzamidine moiety. In **21**, the direction of spacer is inverted. Compounds **13**–**21** bear different residues, either at the 6 or 7 position, with fluorinated aryl groups as a typical substructure present in **13**–**18** [39,40,41]. These compounds have also been evaluated towards thrombin, trypsin, factor Xa and at the fibrinogen receptor. Except for **14**, the fluorinated derivatives showed a strong thrombin inhibition and, moreover, **13**, **15** and **16** inhibited thrombin better than trypsin and factor Xa. [39,41]. Compound **18** was also characterized with respect radical scavenging activity, lipid peroxidation of linoleic acid and lipoxygenase inhibition [40]. The last five entries in Table 2 comprise benzamidine derivatives with more dissimilar structures [41,42,43]. Compound **21** has an anilide substructure, **22** and **23** bear other residues than methyl at position 4, and (*R*)-**24** and **25** lack the 3,4-dihydro-2*H*-1,4-benzoxazine scaffold.

**Table 1:** Matriptase-2 inhibition by 4*H*-3,1-benzothiazin-4-ones.

Compd	Structure	IC50 (µM) ± SEM ^a^
**1** [34]	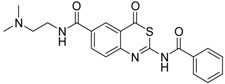	148 ± 28
**2** [34]	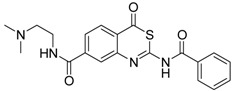	>160
**3** [32]	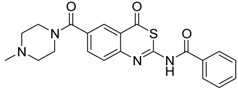	>160
**4** [32]	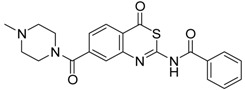	>160
**5** [32]	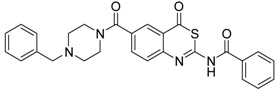	>160
**6** [32]	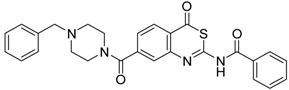	>160
**7** [32]	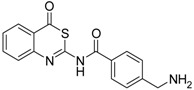	119 ± 9
**8** [32]	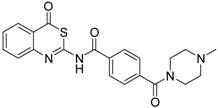	>160
**9** [32]	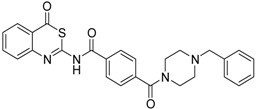	>160

^a^ Values with SEM refer to duplicate measurements with five different inhibitor concentrations. Limits refer to duplicate measurements with a single inhibitor concentration of 40 µM.

**Table 2:** Matriptase-2 inhibition by 2,3-dihydro-1,4-benzodioxines and 3,4-dihydro-2*H*-1,4-benzoxazines.

Compd.	Structure	IC_50_ (µM) ± SEM ^a^
(***S***)**-10** [38]	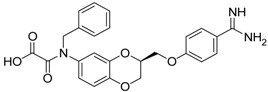	16.7 ± 1.7
(***R***)**-10** [38]	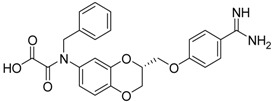	27.3 ± 2.0
(***S***)**-11** [38]	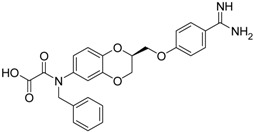	29.8 ± 1.6
(***R***)**-11** [38]	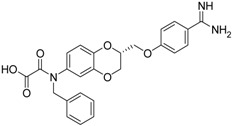	33.6 ± 1.1
(***S***)**-12** [38]	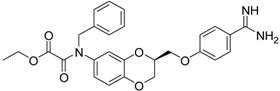	8.47 ± 0.76
**13** [39]	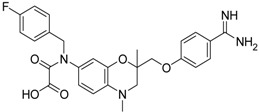	>40
**14** [39]	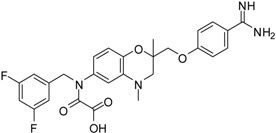	35.8 ± 1.1
**15** [39]	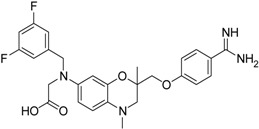	25.8 ± 4.0
**16** [39]	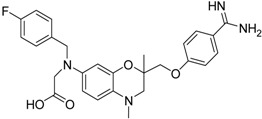	13.6 ± 2.31
**17** [39]	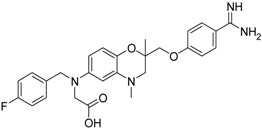	11.2 ± 1.61
**18** [39,40]	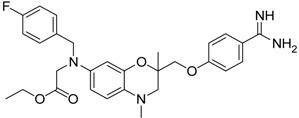	31.6 ± 2.69
**19** [41]	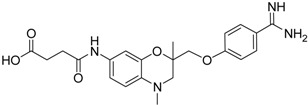	38.8 ± 3.14
**20** [41]	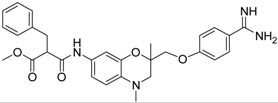	31.4 ± 3.6
**21** [41]	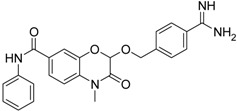	42.6 ± 4.7
**22** [41]	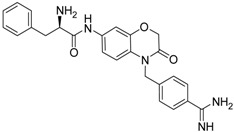	30.9 ± 3.4
**23** [42]	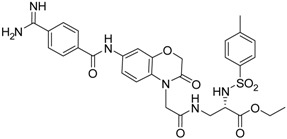	20.5 ± 2.0
(*R*)-**24** [43]	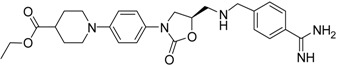	>40
**25** ^b^	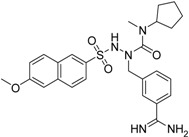	>40

^a^ Values with SEM refer to duplicate measurements with five different inhibitor concentrations. Limits refer to duplicate measurements with a single inhibitor concentration of 40 µM; ^b^ Compound **25** was prepared using a protocol described in reference [44].

**Figure 2:**
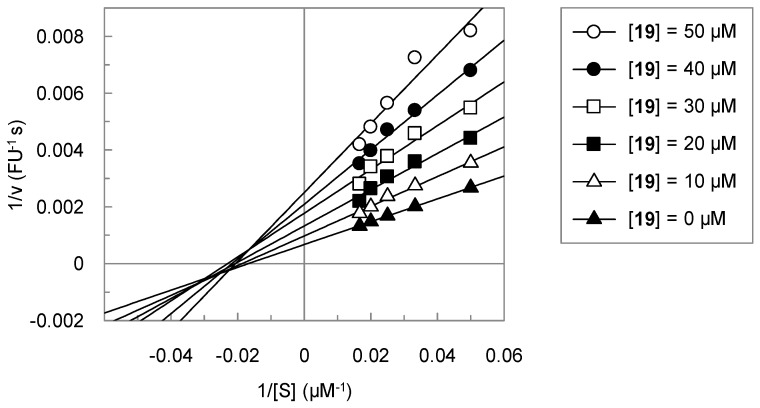
Double reciprocal plot for the inhibition of matriptase-2 by **19**. Substrate concentrations of 20, 30, 40, 50 and 60 µM were used.

In the course of this study, we identified several benzamidine-substituted heterocycles as inhibitors of matriptase-2. For one of these active compounds, **19**, the influence of the substrate concentration on the inhibition was assessed. The Lineweaver-Burk plot is shown in Figure 2. Unexpectedly, compound **19** did not behave as a competitive inhibitor, but showed a mixed type of inhibition.

Among the active compounds, (*S*)-**12** was found to be a potent inhibitor of matriptase-2 with an IC_50_ value of 8.47 µM. A comparison of the activity of the two analogues (*S*)-**12** and (*S*)-**10** revealed a slightly stronger activity of the oxamic ester (*S*)-**12** than that of the oxamic acid (*S*)-**10**. Among the pairs of enantiomers, the (*S*)-configuration was somewhat preferred for matriptase-2 inhibition ((*S*)-**10** *versus* (*R*)-**10** and (*S*)-**11** *versus* (*R*)-**11**). It should be noted that (*S*)-**12** was described to be a highly potent thrombin inhibitor [38]. However, thrombin inhibition is not always accompanied by matriptase-2 inhibition. For example, **13** was inactive at matriptase-2, but highly active at thrombin [39]. Among the 3,4-dihydro-2*H*-1,4-benzoxazines with a methyl group at 4-position (**13**–**21**), several members inhibited matriptase-2 with IC_50_ values of less than 30 µM. The presence of an oxamate moiety (in **13** and **14**) appeared to be less favorable. This could be concluded from the results of the inactive compound **13** and of **16** (IC_50_ = 13.6 µM). The higher flexibility of the glycine substructure (in **15**–**17**) compared to the oxamate substructure (in **13** and **14**) might account for this effect. The position of the *N*-substituted glycine moiety, as either 7- or 6-substituent, did not exert a remarkable influence on matriptase-2 inhibition (**16** *versus* **17**).

The common feature of the fluorine-free compounds **19** and **20** is the NHCO group at position 7. Both compounds were moderately active. The 3,4-dihydro-2*H*-1,4-benzoxazine derivatives **21**–**23** did not show an improved inhibitory activity, and (*R*)-**24** and **25** were inactive. The finding that the latter two compounds did not affect matriptase-2 activity indicated that the presence of a benzamidine moiety does not necessarily lead to matriptase-2 inhibition. This was in accordance with the lack of inhibitory activity of benzamidine itself. On the one hand, the absence of the benzo-fused heterocyclic core in (*R*)-**24** and **25** was obviously unfavorable. On the other hand, since the majority of 2,3-dihydro-1,4-benzodioxines and 3,4-dihydro-2*H*-1,4-benzoxazines were active, these scaffolds are suitable for the positioning of various residues and for directing them to the target’s binding pockets.

In summary, representatives of three heterocyclic classes (4*H*-3,1-benzothiazin-4-ones, 2,3-dihydro-1,4-benzodioxines and 3,4-dihydro-2*H*-1,4-benzoxazines) were identified as inhibitors of matriptase-2. The three heterocyclic scaffolds are similar as they consist of a benzene ring fused to a six-membered heterocyclic ring. The results enabled us to assess the effect of certain residues on biological activity. Even though these compounds are not expected to be selective, this set of data can be used for the future design of new compounds in which such residues were placed at different positions at the bicyclic core in a combinatorial way. For example, the 4-benzamidino-oxymethylene group might be introduced into the 4*H*-3,1-benzothiazin-4-one scaffold. The first attempts to decorate the 4*H*-3,1-benzothiazin-4-one heterocycle with a benzamidine moiety failed, because the scaffold was found to be unstable under the conditions used to convert a nitrile to an amidine group. Moreover, the substituents at positions 7 or 6 present in the active compounds (*S*)-**12** and **17** might be introduced into the 4*H*-3,1-benzothiazin-4-one scaffold. The 6-substituent of **1** or the 2-substituent of **7** might also be considered for the design of new members of the 2,3-dihydro-1,4-benzodioxine and 3,4-dihydro-2*H*-1,4-benzoxazine series. Such investigations are planned for the future in our laboratories.

## 3. Experimental Section

### 3.1. Assays for Human Matriptase-2 Inhibition

The conditioned medium of HEK-MT2 cells was used as a source of matriptase-2 activity and assay conditions were as follows [11,19,25]. Assay buffer was 50 mM Tris–HCl, 150 mM NaCl, pH 8.0. The conditioned medium was collected and concentrated, and aliquots of the supernatant were stored at −20 °C. After thawing, it was diluted with assay buffer (1:10 or 1:20 depending on the enzyme activity) and kept at 0 °C not longer than 8 h. The assays were performed at a FLUOstar OPTIMA PlateReader (BMG Labtech, Ortenberg, Germany). A 10 mM stock solution of the fluorogenic substrate Boc-Gln-Ala-Arg-AMC (Bachem, Bubendorf, Switzerland) in DMSO was diluted with assay buffer. The final concentration of the substrate was 40 µM and of DMSO was 6%. The substrate concentration of 40 µM refers to 1.24 × *K*_m_ [19]. Into each well containing 163.8 µL buffer, 11.2 µL of an inhibitor solution in DMSO and 10 µL of a substrate solution (800 µM) were added and thoroughly mixed. At 37 °C the reaction was initiated by adding 15 µL of diluted conditioned medium and followed over 400 s. All measurements were performed in duplicate with a single inhibitor concentration of 40 µM. Active inhibitors were investigated in duplicate with five different concentrations. Benzamidine hydrochloride was purchased from Acros Organics (Geel, Belgium).

### 3.2. Analysis of the Kinetic Data 

Progress curves were analyzed by linear regression. IC_50_ values were determined by nonlinear regression using the equation v_s_ = v_0_/(1 + [I]/IC_50_), where v_s_ is the steady-state rate, v_0_ is the rate in the absence of the inhibitor, and [I] is the inhibitor concentration. Standard errors of the mean (SEM) values refer to this nonlinear regression.

### 3.3. Purity of Tested Compounds

After performing the kinetic measurements, the purity of the compounds was exemplarily checked by LC/MS. The purity was determined by HPLC-UV obtained on an LC-MS instrument (Applied Biosystems API 2000 LC/MS/MS (Darmstadt, Germany), HPLC Agilent 1100 (Waldbronn, Germany). UV absorption was detected from 220 to 400 nm using a diode array detector. In some cases, the DMSO stock solutions which were used for the inhibition assays were directly subjected to LC/MS. Elution was performed with a gradient of water/MeOH either containing 2 mM ammonium acetate from 90:10 up to 0:100 for 10 min at a flow rate of 300 μL/min. The compounds (*S*)-**10**, **13**, **22**, **23** and (*R*)-**24** showed a purity of more than 90%. Compound **25** showed a purity of 81%. In case of compounds, from which stock solutions were prepared immediately before the kinetic measurements were performed, purity was checked as follows. Solutions in DMSO (**1**–**6**) or acetonitrile (**7**, **8**) were prepared and subjected to LC/MS. Elution was performed with a gradient of water/MeOH either containing 2 mM ammonium acetate from 90:10 up to 0:100 for 10 min at a flow rate of 300 μL/min (compounds **1**–**6**) or with a gradient of water/MeOH either containing 2 mM ammonium acetate from 60:40 up to 0:100 for 10 min at a flow rate of 300 μL/min (compounds **7** and **8**) The compounds **1**, **2**, **3**, **4**, **5**, **6** and **8**, showed a purity of more than 90%. Compound **7** showed a purity of 86%.
